# New Appliances and Things Medical

**Published:** 1902-11-08

**Authors:** 


### NEW APPLIANCES AND THINGS MEDICAL.

**[We shall be glad to receive at our Office, 28 & 29 Southampton Street, Strand, London, W.C., from the manufacturers, specimens of all new preparations and appliances which may be brought out from time to time.]**

### PYRILIN AND IODISED OIL.

(Lorimer and Co., Limited, Britannia Row, London, N.)

Pyrilin is the ethyl phosphurate of pyridin, and is prepared from tar. Pyridin in its various preparations is highly valued as a therapeutic agent in the treatment of bronchitis, asthma, phthisis, and other affection of the lungs. It possesses, however, in its simple form a most disagreeable smell. The ethyl phosphurate of pyridin, in the form in which it is presented in Lorimer's preparation, possesses no disagreeable odour, and it is decidedly an article of pharmaceutical solution. We commend it to the notice of those who fail to obtain satisfactory results in the treatment of chronic diseases of the lungs with the general remedies employed. The iodised oil, manufactured by the same firm, is one of the best forms with which we are acquainted in which iodine can be applied to the skin in free doses without irritation or vesication. It does not stain the skin or liver, and is very rapidly absorbed. For painting large surfaces of the chest, limbs, face, or joints in cases of inflammation, rheumatism, or neuralgia it is invaluable.

### MAGGI'S CONSOMMÉ AND OTHER PREPARATIONS.

(Sole Agents, Cosenza & Co., 95 Wigmore Street, London, W.)

We have more than once favourably mentioned Maggi's Consommé in these columns. It is a beautifully prepared extract of meat with nutritive properties, and most delicately flavoured. Gluten Cocoa is a new preparation which will probably find a valuable place in the dietaries of the sick. It is sold in the form of tablets, which will be found most convenient and economical. The combination of cocoa with gluten supplies what cocoa does not itself contain to any appreciable extent, namely, a nitrogenous element of food. Gluten Cocoa contains representatives of all the important varieties of food, and is therefore a complete food, a nutritive food, and one which is very agreeable to the palate Maggi's Essence we commend to the notice of those who are responsible for the preparation of invalid soups and broths: it is highly concentrated, and greatly improves soups and stocks, imparting to them a delicate and agreeable flavour without introducing any fiery or highly-spiced condiment which is so generally used for flavouring soups, etc., and which have too stimulating an action on the mucous membrane of the stomach and on the action of the liver. Maggi's Cross-star Soups: These soups are contained in packets, and only require to be boiled for a few minutes to be ready for use. They are sold in great variety, and for the most part contain a rich preparation of vegetable substance of high nutritive value.

### DYMAL.

(Zimmer and Co., Frankfurt. London Agents: Bidenmann, Broicher & Co., 33 Lime Street, London, E.C.)

Dymal, which is a dimyn-salicylate, is a very fine, odourless powder, intended to be used as a dry dusting powder or combined in the form of a lanoline ointment. This new powder is non-irritating, possesses an inhibitory action on suppurating processes, and has been found particularly useful in the treatment of acute eczema, in hyperidrosis, simple forms of intertrigo, in bed sores, and in senile gangrene. Dymal is a cheap powder and therefore possesses advantages over many of the antiseptic dressing-powders in general use.

### EXTRACT OF PINE.

(Health Resorts Bureau, 27 Shoe Lane, London, E.C.)

This variety of extract of pine is a carefully manufactured preparation made from the cones of pinus pumilio. It is specially recommended for home use. When pine baths are found of service, great expense and trouble are saved by adding this pure preparation of the resinous extract to the water.

